# Keratinocyte Exosomes for Topical Delivery of Tofacitinib in Treatment of Psoriasis: an *In Vitro/ In Vivo* Study in Animal Model of Psoriasis

**DOI:** 10.1007/s11095-023-03648-0

**Published:** 2024-01-23

**Authors:** Pouya Dehghani, Jaleh Varshosaz, Mina Mirian, Mohsen Minaiyan, Mohammad Kazemi, Mahdi Bodaghi

**Affiliations:** 1 Novel Drug Delivery Systems Research Center, Department of Pharmaceutics, Faculty of Pharmacy, Isfahan University of Medical Sciences, PO Box 81745-359, Isfahan, Iran; 2 Department of Pharmaceutical Biotechnology, School of Pharmacy, Isfahan University of Medical Sciences, Isfahan, Iran; 3 Department of Pharmacology, School of Pharmacy and Pharmaceutical Sciences, Isfahan University of Medical Sciences, Isfahan, Iran; 4 Department of Genetics and Molecular biology, School of Medicine, Isfahan University of Medical Sciences, Isfahan, Iran; 5 Reproductive Sciences and Sexual Health Research Center, Isfahan University of Medical Sciences, Isfahan, Iran; 6 Department of Engineering School of Science and Technology Nottingham Trent University, Nottingham, NG11 8NS UK

**Keywords:** keratinocyte exosomes, Janus kinase inhibitor, psoriasis, targeted delivery, tofacitinib

## Abstract

**Introduction:**

Exosomes are extracellular vesicles in the range of 40-150 nm released from the cell membrane. Exosomes secreted by keratinocytes can communicate with other keratinocytes and immune cells with specific biomarkers at their surface, which may be effective on inflammation of psoriasis and its pathogenesis.

**Objective:**

The present study aimed to formulate and study effectiveness of an exosomal delivery system of tofacitinib (TFC).

**Methods:**

TFC was loaded by different methods in exosomes and then characterized for particle size, zeta potential, drug loading efficiency, and release efficiency. By comparing these parameters, the probe sonication method was chosen to load TFC into exosomes. The MTT assay was used to compare the cytotoxicity of the free drug with the TFC-loaded exosomes (TFC-Exo), and Real-time PCR was used to determine the expression levels of several genes involved in psoriasis expressed in the A-431 keratinocyte and their suppression after treatment. Animal model of psoriasis was induced in BALB/c mice by imiquimod and the efficacy of free TFC, and TFC-Exo were studies on macroscopic appearance and histopathological symptoms.

**Results:**

Exosomes encapsulating TFC showed lower cytotoxicity in MTT assay, higher suppression the expression of *TNF-a, IL-23, IL-6*, and *IL-15* genes in real-time PCR and better therapeutic effect on animal models compered to free TFC.

**Conclusions:**

This method of drug delivery for TFC may be effective on enhancing its therapeutic effects and reduction its side effects favorably in chronic administration.

**Graphical abstract:**

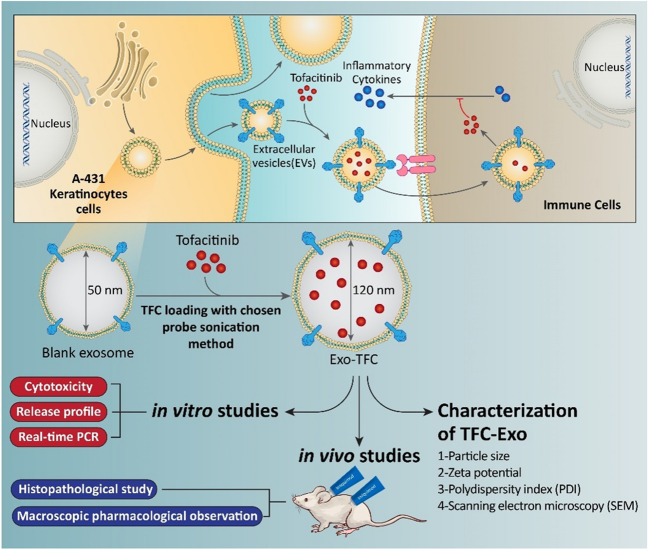

## Introduction

Two percent of the global population suffers from an autoimmune disease named psoriasis. Being chronic, damaging, destructive, and incapacitating are just a few issues that patients with this disease confront. This illness is often associated with the skin, although it may also affect other organs [1]. Psoriasis is characterized by epidermal hyperplasia followed by chronic inflammation caused by the abnormal and uncontrolled growth of keratinocytes. Dendritic cells, macrophages, T cells, and neutrophils invade during inflammation. Topical therapy is one of the most effective treatments for patients with mild to moderate skin conditions. However, because the illness is chronic, treatment must be continuous, and the difficulties posed by currently available drugs, particularly their undesirable side effects, must be considered [2, 3].

Various inflammatory pathways cause psoriatic plaques and their associated symptoms. Therefore, different medications have multiple effects on psoriasis plaques [4]. For example, plaque-type psoriasis may be associated with the *TNF-IL-23-Th17* inflammatory pathway. Consequently, drugs targeting *TNF, IL-23*, and IL-17, as well as signaling pathways such as *JAK/STAT*, can help to treat plaque of psoriasis [5]. Tofacitinib (TFC) is an inhibitor of JAC1 and JAC3. In T cell-stimulated mouse models, TFC inhibits the expression of *IL-17F, IL-17A, IL-23, IL-22, IL-6*, and *TNF-α*. By blocking *IL-23*, the *IL-23/Th17* axis is suppressed, and this drug is anticipated to be beneficial against disorders such as psoriasis. Additionally, TFC citrate blocks *IL-15*, which boosts its production in psoriatic lesions and improves keratinocyte resistance [6, 7].

Extracellular vehicles (EVs) are used for cell-to-cell communication. Exosomes are essential extracellular vesicles with a structure of two spherical lipid layers at the nanoscale, with a density of 1.13-1.19 g/mL and a diameter between 40 and 100 nm. It has been established that various cell types (keratinocytes, fibroblasts, melanocytes, and immune cells) exchange information via these vesicles in skin tissue [8]. Exosome membranes include a variety of proteins and nucleic acids, the specifics of which depend on the cell type from which they are shaded. These proteins play vital roles in recognizing sites on target cells and in facilitating communication with nearby or distant cells. Recent research works have focused on identifying the role of exosomes in the pathogenesis and clinical presentation of autoimmune diseases such as psoriasis. In the pathogenesis of psoriasis, exosomes derived from keratinocytes mediate communication between keratinocytes, innate immunity, and specific immunity [9].

A study comparing the rates of infiltration of keratinocyte-derived exosomes into immune cells with those of non-keratinocyte-derived exosomes discovered that keratinocyte-derived exosomes aggregated more densely in immune cells [10]. Nevertheless, research on the potential of exosomes as drug vehicles has progressed. Because of their liposome-like structure, exosomes in aqueous solutions, creams, and water-in-oil emulsions have a high potential to penetrate the keratinocyte layer of the skin. Conventional vehicles in skin drug delivery, such as liposomes, have similar capabilities; however, exosomes have advantages such as less cytotoxicity, followed by biocompatibility owing to the same source of extraction and site of application, and benefits from specific biomarkers that induce them to fuse with the target cell membrane. This can cause a reduction in therapeutic dosage, in addition to dense and focused accumulation of the drug and an increase in its concentration in the target cell, with minimal interference to other cells and fewer adverse effects from continuous drug usage [11].

Keratinocyte-derived exosomes have been demonstrated to have a higher tendency to infiltrate neutrophils, and the major site of neutrophil proliferation in inflammatory conditions such as psoriasis is around the stratum corneum, making these exosomes easily accessible to neutrophils [10]. Specific biomarkers on the surface of exosomes have additional properties too. It has been proven that CD9 protein exists as a biomarker on the surface of keratinocyte-derived exosomes, which actively pulls molecules with a positive surface charge such as TFC into the exosome. It can also enhance loading efficiency as a determining parameter in these exosomes compared to liposomes and other conventional carriers [12]. Therefore, we hypothesized that keratinocyte-derived exosomes loaded with immunosuppressive drugs such as TFC may readily and effectively target the delivery of their cargo to cells that induce psoriasis inflammation, including immune cells more effectively than free TFC.

Imiquimod (IMQ) is used to produce psoriatic-like symptoms in mouse model skin. One of the most common strategies for causing psoriatic symptoms is the application of 5% IMQ cream to the animal's ears or shaved backs. TLR7 and TLR8 ligands, as well as the IL-17/23 axis, play critical roles in inducing psoriatic-like symptoms in mice when IMQ cream is topically applied. This approach produces symptoms that are remarkably similar to psoriasis plaques in humans, such as scaling, increased hardness of the epidermis (acanthosis and parakeratosis), neo angiogenesis, and inflammatory infiltration of T cells, neutrophils, and dendritic cells. However, after using this cream, the condition that forms similar to psoriasis, will not be chronic and stable and will not have the typical side effects of psoriasis, such as arthritis [13]. This study aimed to formulate an exosome based delivery system for TFC, an immunosuppressive drug, to create an efficient and targeted treatment approach for IMQ induced psoriasis animal model by employing one of the causative mechanisms of the disease [11] with the hypothesis of more effectiveness of keratinocyte derived exosomes loaded with TFC than free drug.

## Materials and Method

### Materials

Penicillin, and streptomycin were purchased from (Vivacell, Iran). 3-(4,5-dimethylthiazol-2-yl)-2,5-diphenyltetrazolium bromide (MTT) and dialysis bag with 12 kDa cut-off, were supplied by (Sigma, USA), fetal bovine serum (FBS), Roswell Park Memorial Institute medium (RPMI), dimethyl sulfoxide (DMSO) and trypsin were from (BioIDEA, Iran). The exosome isolation kit (EXOCIB) was provided by (Cibbiotech Co., Iran) and the Bicinchoninic acid (BCA) protein assay kit was produced by (DNAbiotech Biotech Co., Iran). Total RNA Extraction Kit was from (Parstous, Iran), cDNA Synthesis Kit from (BIOFACT, Korea) and Real-time PCR master mix 2x was provided by (Eppendorf, Germany). 5% IMQ cream was from (Aldara, MEDA Pharmaceuticals, Solna, Sweden) and betamethasone 0.1% cream was provided from (Aburaihan Pharma Co., Iran). TFC citrate was kindly donated by (Nanoalvand Co, Iran). All other chemicals, solvents, and reagents utilized were of commercial quality.

### Cell Culture

The A-431 human epidermoid carcinoma cell line was cultured in 75 cm^2^ flasks (Iwaki, Japan) in RPMI media supplemented with 10% FBS and 1% penicillin/streptomycin (10,000 U/mL) solution. All cells were kept at 5% CO_2_ at 37°C, with a 95% humidity. The medium was changed every two or three days, and after reaching optimal confluence, it was sub-cultured. A-431 is an adherent, keratinocyte type cell line. In this study, in addition to exosome extraction reference cells, cytotoxicity studies and Real-time PCR were also performed on the same cell line.

### Isolation and Quantitation of Exosomes

Exosomes were isolated from the A-431 cell media by using EXOCIB isolation kit (containing reagents A and B). This kit is designed based on the polymer precipitation method. Cultivation of cells in a serum-free medium is unavoidable to isolate exosomes of that cell line in pure form. Because FBS itself contains large amounts of proteins and cell carriers that remain with the exosomes in the following stages of exosome isolation and impair the results. To remove the serum from the culture medium, the cells were seeded into 75 cm^2^ flasks and incubated in the complete culture medium until cell density reached an average of 80% confluence. In the next step, the entire contents of the medium were aspirated and then washed twice with 15 mL of PBS. Then, 20 mL of the serum-free medium was added at 37°C to the flask and placed it in the incubator for 6 to 14 hours. The duration depends on the tolerance of the cells [14]. After completing these steps, the cell fluid was centrifuged (Hettich, Germany) for 10 minutes at 3000 rpm to remove cellular debris and the supernatant was collected. Next, the reagent A, from the EXOCIB kit, was diluted 5:1 with supernatant, stirred for 5 minutes, and left to incubate at 4°C overnight. Then, after 40 minutes of centrifugation at 3000 rpm, the pellet was washed, and the exosomes was re-suspended in of reagent B (100 μL) on a new plate. Finally, the extracted exosomes were quantified by their total protein content using a Bicinchoninic Acid (BCA) protein assay kit.

### Preparation Methods of Exosomes Loaded with TFC (TFC-Exo)

In this study, five methods were evaluated for loading TFC in exosomes and preparing TFC-Exo: *i)* TFC incubation with donor cells of exosomes, *ii)* TFC incubation with exosomes, *iii)* freeze-thaw cycles, *iv)* probe sonication, and *v)* ultrasonic bath.

In the first method i.e., TFC incubation with donor cells of exosomes after 48 hours of incubation with donor cells in a medium containing TFC at a concentration of 500 μM (less than the IC_50_ of the free drug in 48 hours), we isolated the exosomes using the protocol described in the previous section. Diluting the solution with PBS brought the total protein content down to 1.5 μg/mL.

For the second method i.e., TFC incubation with exosomes method, 2 mL of exosomes purified in PBS at a concentration of 1.5 μg/mL (based on total protein) was mixed with TFC at 37°C until a drug-saturated solution (8.3 mg/mL) was formed, and then the mixture was incubated for 24 hours.

In the third method or freeze-thaw cycle method, 2 mL of saturated solution of TFC in PBS was incubated with an exosome solution at a concentration of 1.5 μg/mL based on total protein for 30 minutes, and then the mixture was frozen at -80°C before bringing it back to room temperature. The freeze-thaw cycle was repeated three times.

In the fourth method or probe sonication method the TFC-saturated PBS solution containing 1.5 μg/mL (based on total protein) was first sonicated using probe sonicator (500 v, 2 kHz, 20% power, 6 cycles by 4 sec pulse /2 sec rest) (Bandelin, HD 3200, Germany), cooled on ice for 2 minutes, and then sonicated again using a probe sonicator. The solution was then incubated at room temperature for 1 hour to allow the exosomes membrane to recover its original properties.

In the fifth method or ultrasonic bath method, after preparing TFC-saturated PBS solution containing 1.5 μg/mL (based on total protein), the mixture was placed in a sonicating bath (Hawashin 505, Korea) for 20 minutes before being incubated at room temperature for 1 hour.

Across all five approaches of drug loading, to isolate the TFC-Exo the resulting solution was centrifuged with an Amicon® filter tube (cutoff 10 kDa, Millipore, Irland) at 7000 rpm for 30 minutes. The Amicon® filter tube was then filled with 2 mL of PBS, and after pipetting and completely distributing the TFC-Exo, the mixture was centrifuged again for 30 minutes at 7000 rpm, so that the newly made exosomes were completely washed and the free drug or bonded drug to the exosome's exterior layer, which leads to the explosive release of the drug, were completely eliminated. At last, we suspended TFC-Exo in the appropriate amount of PBS for further applications.

### Particle Size, Polydispersity Index (PDI) and Zeta Potential of TFC-Exo

Blank exosomes and TFC-Exo were characterized for their zeta potential, the hydrodynamic diameter (reported as intensity-based z-average), and polydispersity index (PDI) using dynamic light scattering method (DLS) by a Zetasizer (Zetasizer 3600, Malvern Instrument Ltd, Worcester, UK). Malvern software (Zetasizer Ver. 7.11) was used to analyze the data. The concentration of exosomes in the solution for use in the DLS technique should be in a range that is not so dilute that the device cannot correctly detect the required indicators and not so thick that it interferes with the dynamic light scattering. By comparing the different concentrations of the exosome solutions diluted with deionized water at room temperature, a 1:1000 dilution ratio was used for measurements, which caused better homogenization of the dispersed phase. It should be noted that zeta potential in deionized water significantly impacts both the magnitude, and at times, the direction (+/-) of the charge on particles.

### Scanning Electron Microscopy (SEM)

The morphological characteristics of the exosomes surface were analyzed using a scanning electron microscope (Leo 1430 VP scanning electron microscope, Germany) operating at a voltage of 26.0 kV. On a clean glass slide, 5 μL of PBS-diluted exosomes were dried at room temperature. It was then examined using the scanning electron microscope.

### TFC Loading Efficiency in Exosomes

After preparation, the TFC-Exo dispersion was added to an Amicon ultra filter tube (10 kDa cut-off) and centrifuged for 30 min at 10,000 rpm to obtain TFC-Exo pellets. Then, for washing, 1 mL of PBS was added to the filter again and pipetted until the exosomes were well mixed in PBS, followed by washing, 1 mL of PBS was pipetted through the Amicon filter until the exosomes were well mixed in PBS, and the centrifugation process was repeated. The collected liquid was divided and quantified by UV spectrophotometry at λmax =285.8 nm (UV-mini-1240, Shimadzu, Kyoto, Japan). TFC standard solutions in PBS were prepared at concentrations ranging from 5 to 50 μM and the calibration curve equation (y = 0.033x+0.0004) (R^2^ = 0.9992) was used to calculate the drug loading efficiency (LE%) which is found by taking the difference between the drug initially used (mg) and the free remaining drug (mg) and dividing the result into the drug initially used (mg) multiplied by 100.

1 $$\textrm{LE}\%=\frac{\textrm{Drug}\ \textrm{initially}\ \textrm{used}\ \left(\textrm{mg}\right)-\textrm{free}\ \textrm{remained}\ \textrm{drug}\ \left(\textrm{mg}\right)}{\textrm{Drug}\ \textrm{initially}\ \textrm{used}\ \left(\textrm{mg}\right)}\times 100$$

### *In Vitro* Drug Release from TFC-Exo

The drug release profiles of TFC-Exo obtained from different drug loading methods were studied. PBS (1 mL) was added to Amicon filters containing blank exosomes and TFC-Exo followed by pipetting the mixtures carefully to create exosomal dispersions. Then TFC-Exo (300 μL) dispersion was transferred to a dialysis bag with a cut-off of 12 kDa (Sigma, USA), and blank exosomes (300 μL) were used also as the blank. The dialysis bag was transferred into PBS (14 mL, 0.05 M, pH 7.4) as the release medium and stirred at 37 °C at a speed of 150 rpm. At 0, 1, 2, 4, 6, 8, 12 and 24 h, an appropriate volume of the release media was removed, the absorbance was measured using a spectrophotometer at λmax=285.5 nm, and the sample was then replaced within the dialysis medium The experiment was performed three times to ensure accuracy. Formulas for calculating the percentage of TFC-Exo released over a 24-hour (RE24%) period from various preparation methods and from a cold cream base loaded with various concentrations of TFC-Exo over a 48-hour (RE48%) period are provided below as Eq. (2).

2 $$RE\%=\frac{\int_0^t{y}_{t\times dt}}{y_{100\times t}}$$

where *y* is the percentage of TFC that has been released over the course of time *t* (here 24 hours and 48 hours), represented as a percentage of the curve that was reached at the point of maximum release, *y* 100, during the same time period. To figure out the integral of the numerator, either the trapezoidal method or the area under the curve was used.

### Preparation of Cold Cream from TFC-Exo for Animal Studies

For preparation of TFC-Exo 2% cold cream, 1.55 mL of TFC loaded exosomes dispersion was mixed with 200 mg cold cream base and for preparing the TFC-Exo 1% in cold cream base 50 mg of the prepared 2% cream was diluted with cold cream to 100 mg. For animal studies TFC or TFC-Exo was mixed in cold cream base. Four samples of free TFC 1% in cold cream base, free TFC 2% in cold cream base, TFC-Exo 1% in cold cream base, TFC-Exo 2% in cold cream base, and blank cold cream were prepared to test the *in vitro* release of TFC using a Franz diffusion cell. In this method, PBS (5 mL, 0.05 M, pH 7.4) was used as the release medium in the receptor chamber and was stirred at 37°C at a speed of 150 rpm. A pre-soaked synthetic cellulose acetate membrane was used as a skin simulator in the cell and each cold cream formulation (10 mg) was placed over the membrane surface. Samples were withdrawn and replaced with fresh receiving medium at 0.5, 1, 2, 4, 6, 8, 12, 24, and 48 hours. As previously mentioned, the samples were spectrophotometrically analyzed for drug content. The procedure was replicated three times.

### *In Vitro* Cytotoxicity Assay

The A-431 cells were grown in a 5% CO_2_, 95% humidity atmosphere at 37°C for 24 hours at a cell density of 5×10^4^ cells/well in 96-well plates. Next, the cells were exposed to a range of concentrations (5-2000 μM) of free TFC and TFC-Exo at 37°C. Subsequently, to one plate after 24 hours and to another one after 48 hours, 20 μL of MTT (5 mg/mL) solution was added, and cells were incubated for 4 h. Finally, the medium containing MTT was replaced with DMSO (150 μL), and after 30 min, the formazan crystals were dissolved in DMSO, and determined using a 570-nm wavelength to measure absorbance in an ELISA microplate reader (Hiperion, Germany).

### Real-Time Reverse Transcription PCR Assay

Two 6-well plates were used to cultivate A-431 cells at a density of 5×10^5^ cells/well, and then incubated for 24 h at 37°C and 5% CO_2_. Then, both free TFC and TFC-Exo were added to a medium containing TFC (50 μM) in three wells. Blank exosomes were added to the other 3 wells with the exosome concentration equivalent to the wells containing TFC-Exo. The remaining three wells were left untreated as control untreated cells. The adherent cells were collected 24 hours later. Total RNA was extracted using Total RNA extraction kit (ParsTous, Iran) according to the manufacturer instruction. 1 microgram of RNA was reverse transcribed using the BioFact™ 2X RT Pre-Mix kit (BIOFACT, Korea) with Oligo-dT primer. Real-Time PCR analysis was performed on the Mic qPCR instrument (Bio Molecular Systems, Australia) using gene specific primers (Table I) and Ampliqon-RealQ Plus 2x Master Mix Green (Ampliqon, Denmark). Glyceraldehyde 3-phosphate dehydrogenase (*GAPDH*) was used as housekeeping gene. The PCR amplification settings were 15 min at 95°C, followed by 40 cycles of denaturation for 20 sec at 95°C, annealing for 30 sec at 60°C, and extension for 30 sec at 72°C. Melting curve analysis was used to determine specificity of PCR products. All qRT-PCR experiments were performed in triplicate. The relative gene expression was measured using the 2^−ΔΔCt^ method as described previously [15].

**Table I:** Primers Sequences for Real Time PCR

Gene Name	Primer Sequences (5′→3′)	Accession Number	Ampliqon Size (bp)
*TNF-α*^*a)*^	F: CCCAGGGACCTCTCTCTAATC	84	NM_000594.4
	R: ATGGGCTACAGGCTTGTCACT		
IL23A^b)^	F: GAGCCTTCTCTGCTCCCTGATA	121	NM_016584.3
	R: GACTGAGGCTTGGAATCTGCTG		
*IL-6*^*c)*^	F: AGACAGCCACTCACCTCTTCAG	132	NM_000600.5
	R: TTCTGCCAGTGCCTCTTTGCTG		
*IL-15*^*d)*^	F: AACAGAAGCCAACTGGGTGAATG	148	NM_172175.3
	R: CTCCAAGAGAAAGCACTTCATTGC		
*GAPDH*^*e)*^	F: GTCTCCTCTGACTTCAACAGCG	131	NM_002046.7
	R: ACCACCCTGTTGCTGTAGCCAA		

a) Tumor necrosis factor alpha; b) Interleukin-23 subunit alpha; c) Interleukin-6 receptor; d) Interleukin-15; e) Glyceraldehyde 3-phosphate dehydrogenase

### Animal (*in vivo*) Studies

#### Treatment Groups

**Fig. 1:**
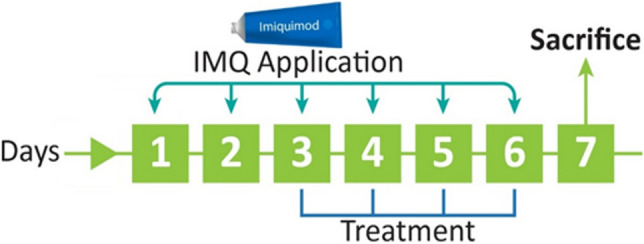
Infographic summary for the treatment of psoriasis-like symptoms induced in BALB/c mice by imiquimod (IMQ). IMQ was topically applied from Day 1 to Day 6 and different treatment were applied from Day 3 to Day 6. The animals were sacrificed on day 7.

45 male adult BALB/c mice, weighing 18-22 g, aged 6-8 weeks, were kept in a ventilated room with unrestricted access to food and water during a light/dark cycle of 12 hours. The guidelines for animal research established by the Isfahan University of Medical Sciences ethical committee were followed (ethical code: IR.MUI.RESEARCH.REC.1399.589) and they were conducted according to the NIH Guide for the Care and Use of Laboratory Animals. The whole duration of the experiment was 7 days. The mice were randomly placed into nine following groups in preventive treatment mode, each with five animals. Initially, the animals’ back was shaved (2×3 cm) at first by a razor and then by a depilatory cream. Throughout the experiment, the normal group received no therapy. In the remaining groups over the duration of the trial, from Day 1 to Day 6, imiquimod (IMQ) 5% cream was applied on the left ear (20 mg/day) and on the shaved back of the animals (62.5 mg/day) [16]. Except for the Untreated psoriatic control group, the remaining groups were treated two hours before applying the IMQ from the third to the sixth day of the experiment (Figure 1). The blank cold cream group received cold cream on the left ear and shaved back (3 and 9 mg/day, respectively) in order to evaluate the impact of the blank cream. The animals in the Blank exosomes in a cold cream group were treated with blank exosomes in a cold cream base on the left ear and shaved back (3 and 9 mg/day, respectively). The concentration of exosomes in the cold cream was the same as the concentration of exosomes in the TFC-Exo 2% in the cold cream base. TFC 1% in the cold cream group and TFC 2% in the cold cream group received TFC 1% and 2% in the cold cream base on the left ear and shaved back (3 and 9 mg/day, respectively), whereas TFC-Exo 1% in the cold cream group and TFC-Exo 2% in the cold cream group received TFC-Exo 1% and 2% in the cold cream base on the left ear and shaved back (3 and 9 mg/day, respectively). As a reference treatment, the betamethasone 0.1% cream group received betamethasone 0.1% cream on the left ear and shaved back (3 and 9 mg/day, respectively). 24 hours after the last treatment, all animals were sacrificed by inhaling CO_2_. Mice were sacrificed, and samples were taken from the left ear skin lesions, which were then fixed in 10% buffered formalin, washed, dehydrated, and cleared in xylene followed by paraffin embedding.

#### Scoring of Skin Inflammation Severity

On days 1-7 of the trial, the psoriasis area severity index (PASI), which is a modified human grading system, can be used to measure and analyze the severity of inflammation in animal models. The same investigator separately scored thickening, scaling, and erythema on a 0–4 scale: 0, none; 1, mild; 2, moderate; 3, marked; and 4, obvious. As a measure of the level of inflammation, trend lines were plotted and computed an accumulation score [17].

#### Measurement of Ear Thickness

On days 1-7 of the trial, an independent investigator blinded to the treatment groups assessed the ear thickness in triplicate using an electronic micrometer (Louisware, UK). An appropriate indicator of skin irritation is an increase in ear thickness [18].

#### Histopathology and Immunohistochemistry

Paraffin-embedded samples were sectioned (5 μm-thick) with using a microtome (Rotary Microtome Model 1212, Leica, Wetzlar, Germany) stained with hematoxylin and eosin (H&E) and Mason's trichrome to analyze pathological lesions and histological markers. CD3 (Cat. No. ab16669; diluted 1:100) was used for immunostaining test. A fluorescence and light microscope were used to evaluate the stained samples. The histopathological changes evaluation included; the amount of hyperkeratosis, parakeratosis, and acanthosis in H&E-stained samples, as well as the amount of tissue collagen in Mason's Trichrome-stained samples. CD3 in immunostained samples showed how many CD8+ and CD4+ T cells were infiltrated the tissue, and were evaluated semi-quantitatively. The results were rated as follows: 0, none; 1, mild; 2, moderate; 3, marked; 4, obvious.

To take pictures of the samples, a typical Zeiss® microscope with a Sony® color video camera was used. Two researchers made at least three separate observations of each of the above traits in each group, for a total of six observations.

### Statistical Analysis

The data were presented as mean±SD. Analysis of variance (ANOVA) and Tukey's multiple comparison tests were used to determine if the groups' differences were statistically significant. A student's t-test was used to compare two groups. In all statistical analysis, significance was defined as a "*p*" value of less than 0.05. The GraphPad Prism statistical software was used for all the statistical analysis.

## Results

### Isolation and Characterization of A-431 Cell Line-Derived Exosomes

**Fig. 2:**
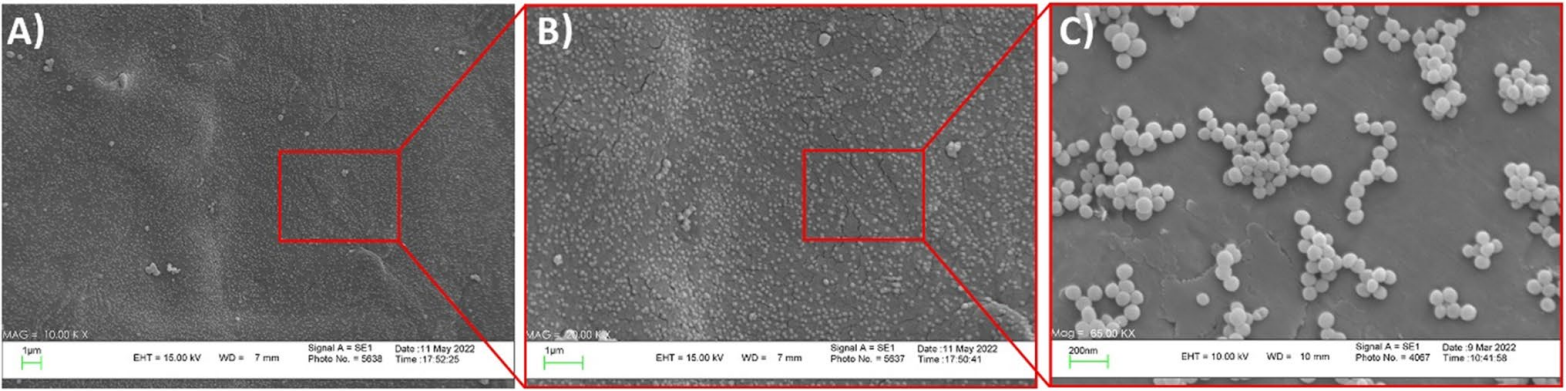
SEM images of A-431 cell line-derived exosomes with different magnifications. Scale bars in (**A**), (**B**) are 1 μm and in (**C**) 200 nm.

Particle size, zeta potential, protein content, and shape were studied in order to describe exosomes isolated from the A-431 keratinocyte cell line. The average particle diameter of blank exosomes was 70.8 ± 2.8 (nm), the average hydrodynamic diameter of blank exosomes was 55.2±12 (nm) with PDI of 0.076±0.02 and zeta potential of -4.8±1.26 (mV) revealed by DLS method (Table II). The spherical surface morphology and highly uniform size distribution of blank exosomes and TFC-Exo created using various methods were verified in SEM imaging (Figure 2). The exosome concentration in the sample was obtained based on the total protein concentration in the sample by the Bicinchoninic acid assay (BCA) method.

**Table II:** Particle Size, PDI and Zeta Potential and Loading Efficiency (LE%) of Blank Exosomes and TFC-Exo Prepared with Different Method (mean ± SD, n = 3). * Shows the Preparation Methods, which are Statistically Significant Different (p<0.05) with each other and with other Methods in each Studied Dependent Variable

Preparation method	Particle size (nm)	Zeta potential (mV)	PDI	LE (%)	RE_24_ (%)
Blank exosomes	55.2±12.0	-4.8±1.3	0.08±0.02	-	-
Incubation donor cell	54.9±10.5	-4.42±0.7	0.08±0.03	0.05±0.03*	64.8±2.3
Incubation exosome	66.0±10.8	-4.59±1.0	0.10±0.02*	1.53±0.11*	62.0±1.6
Freeze-thaw cycle	197.0±113.0*	-3.9±1.2*	0.49±0.20*	13.27±1.35*	59.2±3.1*
Probe sonication	123.5±15.7*	-8.7±1.7*	0.16±0.02*	30.70±1.40*	67.5±2.4*
Ultrasonic bath	96.9±11.7*	-6.7±1.4*	0.08±0.03	14.17±0.80*	62.5±1.8

### Characterization of TFC-Exo

TFC was incorporated into exosomes using different methods: incubation with donor cells, incubation with exosomes, freeze-thaw cycles, probe sonication, and ultrasonic bath. The first two methods are passive. In other words, the membrane structure of exosomes doesn’t need to be reformed to load TFC into them. However, subsequent methods were utilized to induce the deformation /reformation of exosomes in the presence of TFC.

To determine the most efficient method of drug loading in exosomes, the loading efficiency, release efficiency, particle size, zeta potential, and PDI were evaluated and compared before and after drug loading in exosomes (Table II). The mean particle size of blank exosomes and TFC-Exo produced by different methods except for freeze-thaw cycle method, ranged between 55.2±12 (nm) for blank exosomes, and 123.5±15.7 (nm) for TFC-Exo produced by probe sonication method. PDI values changed between 0.076±0.02 for blank exosomes and 0.16±0.02 for TFC-Exo produced by probe sonication method. Except for the freeze-thaw cycle method, which resulted in a significant different (p < 0.05) mean particle size and PDI, the remaining results of mean particle size and PDI were well within acceptable ranges. Like other lipid nano particulate carriers of drugs, the best range of particle size for transdermal delivery is 10-600 nm [19]. The numerical value of PDI ranges from 0.0 (for a perfectly uniform sample with respect to the particle size) to 1.0 (for a highly polydisperse sample with multiple particle size populations). The acceptable range of PDI is 0.0- 0.3 [19].

Table II shows the lowest and the highest absolute values of zeta potential of blank exosomes and TFC-Exo produced by different methods were -3.88±1.24 (mV) for freeze-thaw cycle method and -8.73±1.66 (mV) for probe sonication method that were significantly higher than other methods (p < 0.005). The zeta potential of exosomes prepared by freeze-thaw cycle, probe sonication and ultrasonic bath were significantly different from other methods (p < 0.05). The absolute value of higher zeta potential denotes a stable colloidal system, and this is particularly noticeable for developing drug delivery systems.

Table II shows that the percentage of TFC loading efficiency (LE %) into exosomes increased significantly (p < 0.05) from low to high as follows: Incubation donor cell (the lowest), Incubation exosome, Freeze-thaw cycle, Ultrasonic bath, Probe sonication (the highest).

### *In Vitro* TFC Release Profile Study from TFC-Exo

**Fig. 3:**
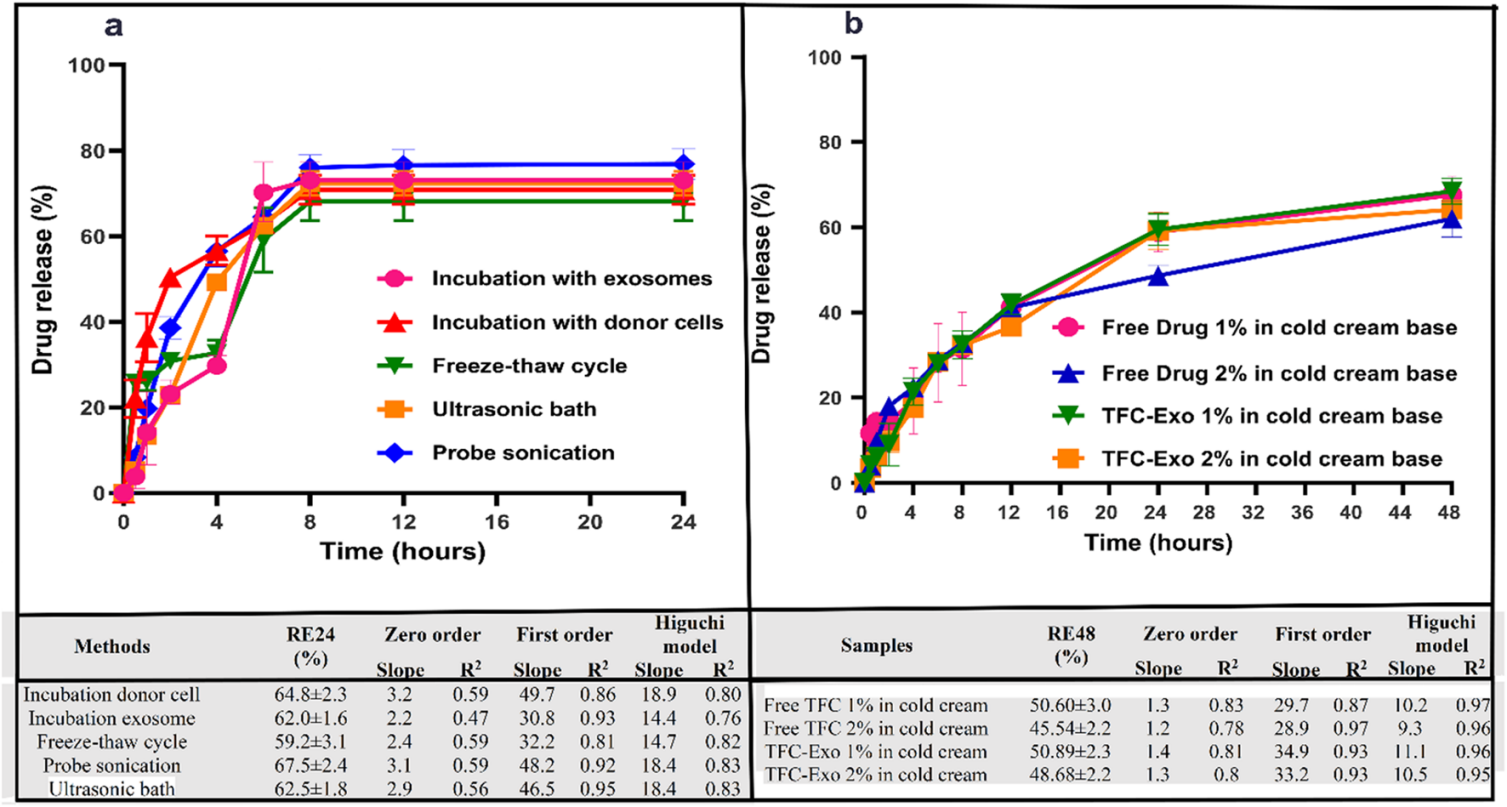
*In vitro* Tofacitinib (TFC) release profiles, RE% and kinetic models of drug release profiles from **a)** TFC-Exo prepared by different methods and **b)** cold cream base loaded with different concentrations of TFC-Exo (results are shown as mean ± SD, n = 3)

The average release efficiency of TFC from loaded exosomes prepared using different methods is shown in Figure 3a. The maximum percentage of drug release efficiency of TFC in 24 hours was observed for TFC-Exo produced via probe sonication (67.5%). In contrast, the lowest was associated with the freeze-thaw cycle method and was 59.2%. The difference between drug release efficiency in the probe sonication method with incubation donor cells, incubation exosomes, and freeze-thaw cycle methods was significant (p < 0.05). However, the differences between the other methods were not statistically significant (p> 0.05). All TFC-Exo preparation methods showed a biphasic release pattern that included a rapid release phase of TFC over the first 8 hours, after which no additional drug release was demonstrated (Figure 3a). A mild burst release was noticeable, especially in the freeze-thaw cycle method (Figure 3a), possibly due to drug entrapment in the exosome membrane that remained even after multiple washes. To apply the exosomes on the skin of animals a cold cream base was used. In the case of psoriasis, formulations with moisturizing properties may be used as adjuvant therapy along with simple cleaning to avoid skin dryness. Oily formulations have been proven to reduce patient compliance. Therefore, a cold cream base was selected between the three traditional formulations of gel, cold cream, and simple ointment because it has sufficient moisturizing capabilities, is less messy, adheres to the skin, and is easier to wash. Figure 3b shows the drug release profiles of TFC from the cold cream formulations, which has considerably slowed down the drug release in comparison with TFC-Exo. As the results shows the release profiles of free TFC and TFC-Exo in the cold cream formulation at the same concentration are extremely similar, but as the drug concentration in the cold cream formulation increased, the drug release percentage reduced significantly (P>0.05).

The kinetic models of drug release profile from TFC-Exo prepared by different methods and from cold cream formulations of TFC-Exo and free TFC were investigated (Figure 3). The kinetic models can predict the mechanism of real phenomena such as drug diffusion or dissolution, erosion, swelling, precipitation, and degradation of the carrier taken into account to the release profiles. In zero-order the release kinetics of an agent is only a function of time, and the process takes place at a constant rate independent of the concentration of the active agent. This relation can be used to determine the drug dissolution from various types of modified release dosage forms. First-order release kinetics states that the variation in concentration with respect to time depends only on the concentration of the active agent remaining in the device. The dosage form follows this profile such as those containing water soluble drug in a porous matrices release the drug that is proportional to the amount of drug released by unit time diminish. Higuchi model studies the release of water soluble and low soluble drugs incorporated in semisolid and solid matrices. This model studies the dissolution from a homogeneous matrix. To study the dissolution from a spherical heterogeneous matrix system, where the release occurs through pores in the matrix, Higuchi describes drug release as a diffusion process based in the Fick’s law, square root time dependent [20]. Based on the *R*^*2*^ of each kinetic model, drug release profile from TFC-Exo, was more matched with the first-order model (Figure 3a). But for cold cream formulations it completely best fitted with the Higuchi model (𝑅^2^ > 0.95) (Figure 3b).

### Cytotoxicity Studied by MTT Assay

**Fig. 4:**
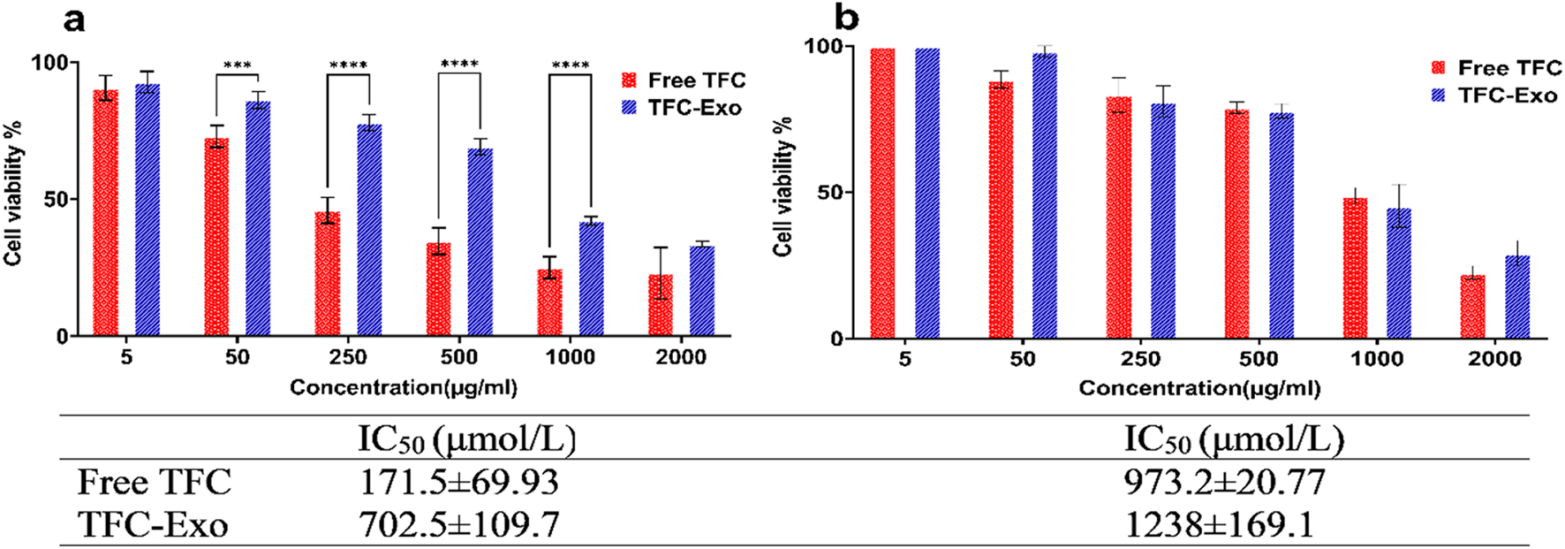
*In vitro* cytotoxicity of different studied treatments on A-431 cells by MTT Assay after **a)** 24 hours and **b)** 48 hours (mean ± SD, n = 3). Significant difference shown by symbols: p < 0.05 (*), p < 0.005 (**), p < 0.0005(***) and, p < 0.0001(****).

In A-431 cells, the percentage of cells viability in free TFC and TFC-Exo are shown for different concentrations of TFC (Figure 4). The 24-hour MTT assay results showed that the percentage of cell viability of the TFC-Exo was significantly higher than the free TFC sample at the same concentrations of the drug, while there was no significant difference in any of the concentrations in the 48-hour results. Also, the IC_50_ values of TFC-Exo after 24 h were significantly higher (p < 0.005) than free TFC (702.5±109.7 μmol/L *vs.* 171.5±69.9 μmol/L), but the difference between the IC_50_ values of TFC-Exo and free TFC in 48 h (1238.0±169.1 μmol/L *vs.* 1238.0±169.1 μmol/L) did not turn out to be statistically significant (p > 0.05) (Figure 4).

### RT-PCR Results

**Fig. 5:**
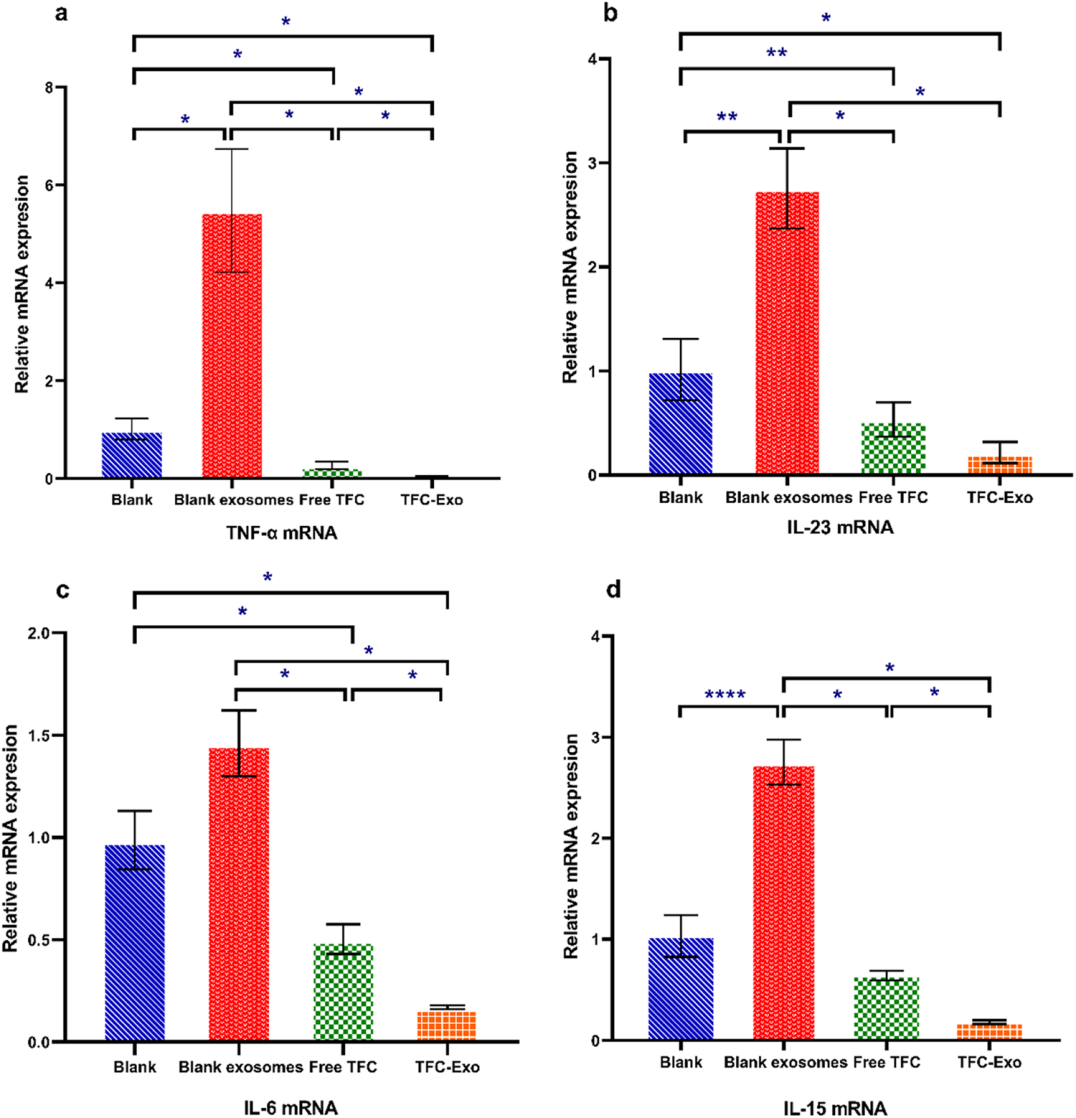
Changes in the expression of the relevant cytokines mRNA of **a)** *TNF-α* mRNA, **b)** *IL-23* mRNA, **c)** *IL-6* mRNA and **d)** *IL-15*, in A-431 cells after treating for 24 hours with blank exosomes, free TFC, and TFC-Exo (50 μΜ) in comparison with control untreated cells (mean ± SD, n = 3). Statistical significances are shown as ^*^p < 0.05, ^**^p < 0.01, ^***^p < 0.001 and ^****^p < 0.0001.

The amounts of mRNA that are expressed for *TNF-α, IL-23, IL-6*, and *IL-15*, which are not only essential cytokines involved in psoriasis but also expressed in the A-431 cell line, were subjected to Real-time PCR in the presence of selected testing groups including; blank exosomes, free TFC, and TFC-Exo (50 μΜ) and control untreated cells for 24 hours. The mRNA expression of all cytokines was significantly different in studied groups (p <0.05) (Figure 5) except *IL-23*, which was similar in free TFC and untreated cells, *IL-6* which was similar in blank exosomes and untreated cells, and *IL-15* that was not different in free TFC and TFC-Exo groups with the untreated cells. In the presence of blank exosomes, the mRNA expression levels of all cytokine genes were significantly upregulated compared to the control untreated cells except *IL-6*. Gene expression for all cytokines was considerably lower in the TFC-Exo group compared to the free TFC group at the same TFC concentration. These data demonstrate that keratinocyte-derived exosomes upregulated the expression of inflammatory cytokine genes in A-431 keratinocytes, but their effects were reversed entirely when treated with TFC-Exo.

### Severity of Animal Skin Inflammation

**Fig. 6:**
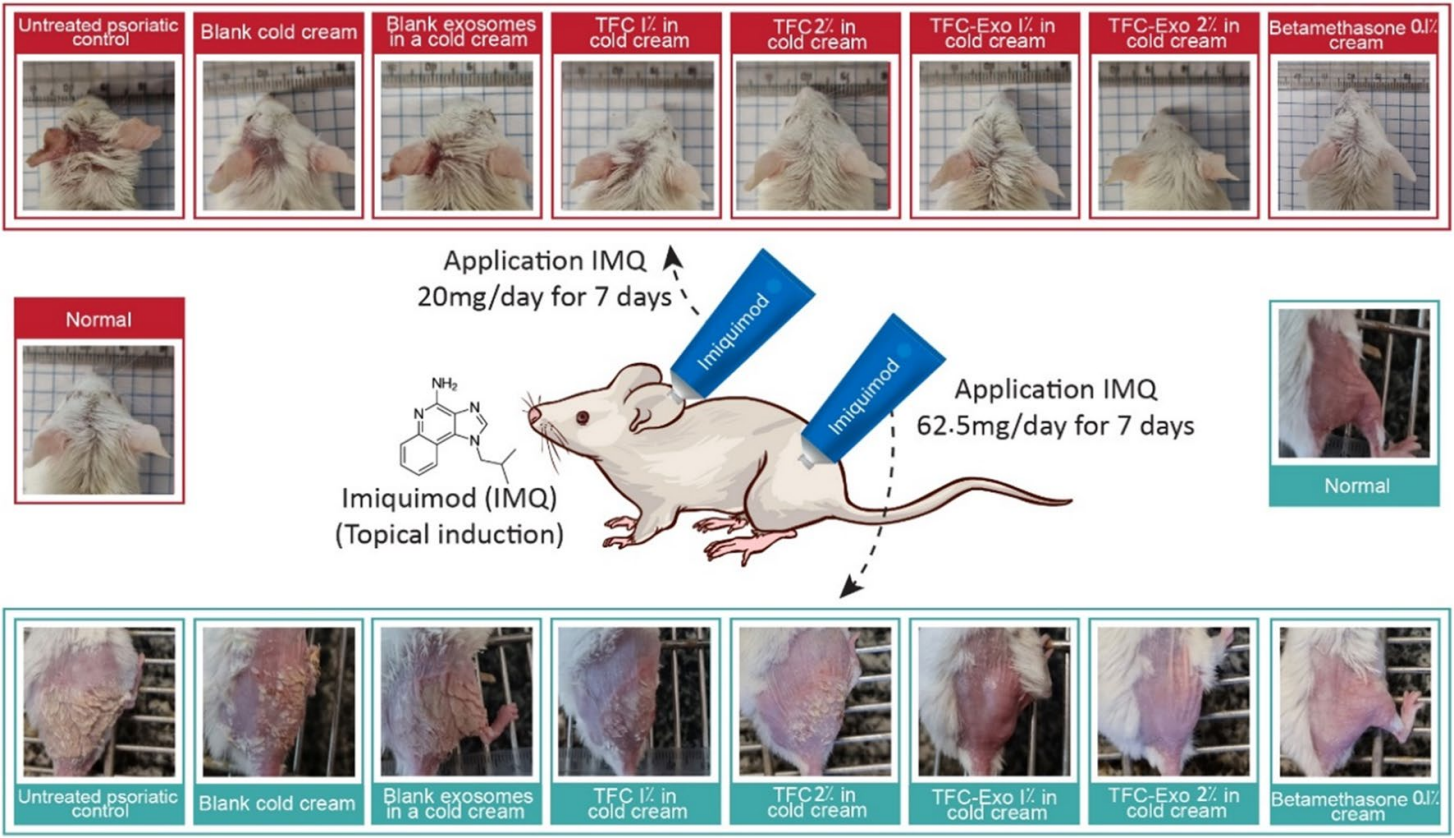
Infographic depicting the usage of IMQ on the back and left ear of BALB/c mouse model, as well as photographs collected on the final day from the ears and back of the mice in each group.

The macroscopic pharmacological observation (Figure 6) was carried out by two methods to evaluate the results on eight experimental groups of animal as psoriatic-like mouse model caused by IMQ with different treatments and a group without using imiquimod as normal group.

**Fig. 7:**
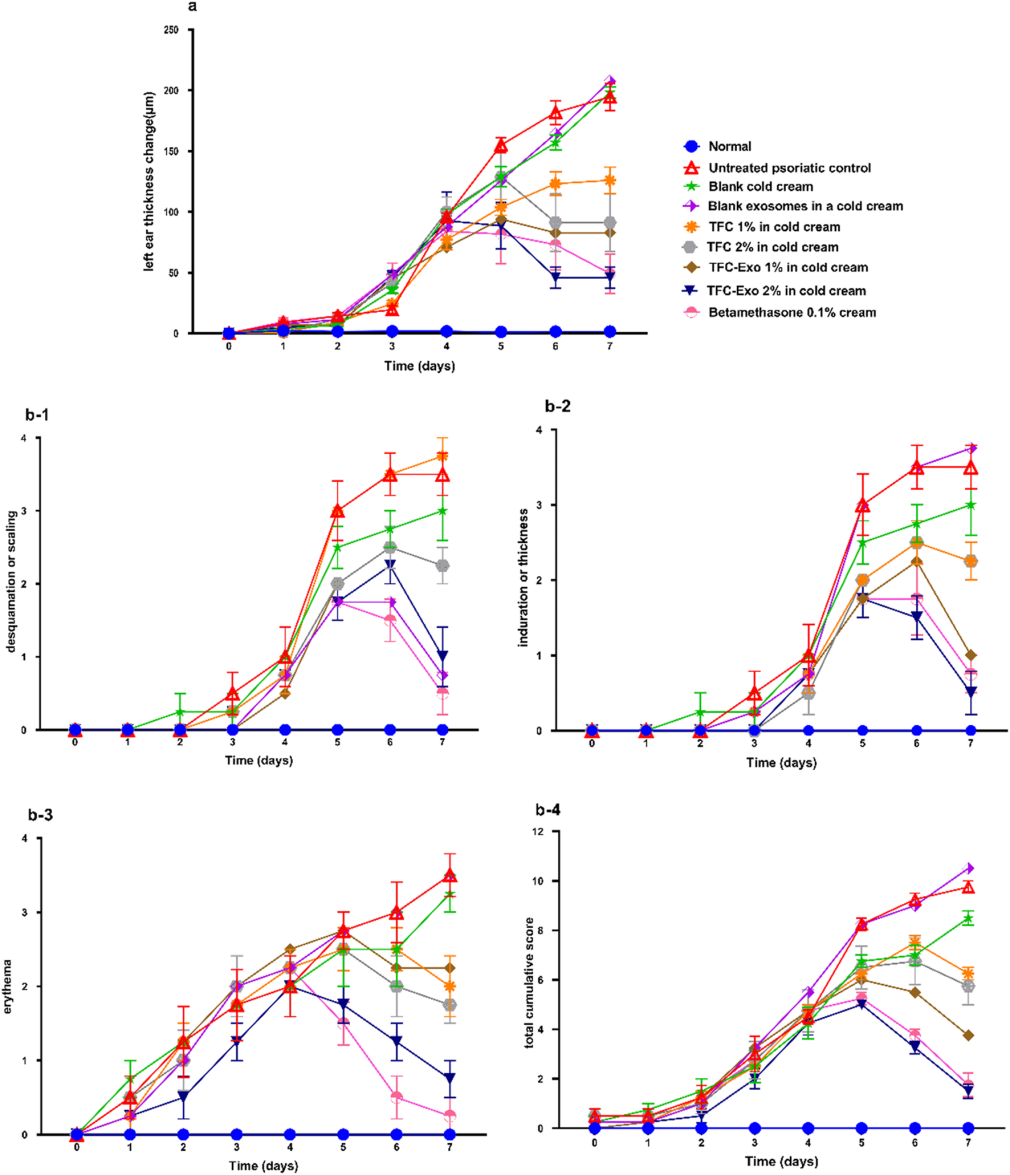
Evaluation of **a)** left ear thickness change compared to baseline and **b)** PASI score of **(b-1)** desquamation or scaling, **(b-2)** induration or thickness, **(b-3)** erythema, and **(b-4)** total cumulative score in 9 experimental groups during 7 days treatment (mean±SD, n=5).

The first method was evaluation of left ear thickness changes compared to the baseline for 7 days of treatment (Figure 7a) and the second method was evaluation of the psoriasis area severity index (PASI), which is a modified human grading system. In this method for each group on days 1 to 7 of the experiment, a score from 0 to 4 is given for 3 parameters of scaling, erythema, and thickening as mentioned in section 2.12.2 (Figure 7b). The area under the curve (AUC) was computed for the left ear thickness changes compared to baseline and the cumulative score graph of PASI score were also calculated (Table III).

**Table III:** Measurement of the Area under the Curve (AUC) of the left ear Thickness Changes Compared to Baseline and the Cumulative PASI Score in Different Groups of Treated Animals

Groups	Cumulative PASI Score AUC	Left ear thickness compered to baseline AUC
Normal	-	10.75±3.5
Untreated psoriatic control	31.4±3.1	572.5±21.1
Blank cold cream	26.6±4.0	529.1±17.1
Blank exosomes in a cold cream	32.4±5.4	549.3±42.0
TFC 1% in cold cream	25.5±0.9	401.5±23.2
TFC 2% in cold cream	25.4±7.6	419.9±55.8
TFC-Exo 1% in cold cream	22.9±3.8	348.1±37.1
TFC-Exo 2% in cold cream	16.1±1.7	308.4±45.9
Betamethasone 0.1% cream	18.9±2.8	335.4±54.3

**Fig. 8:**
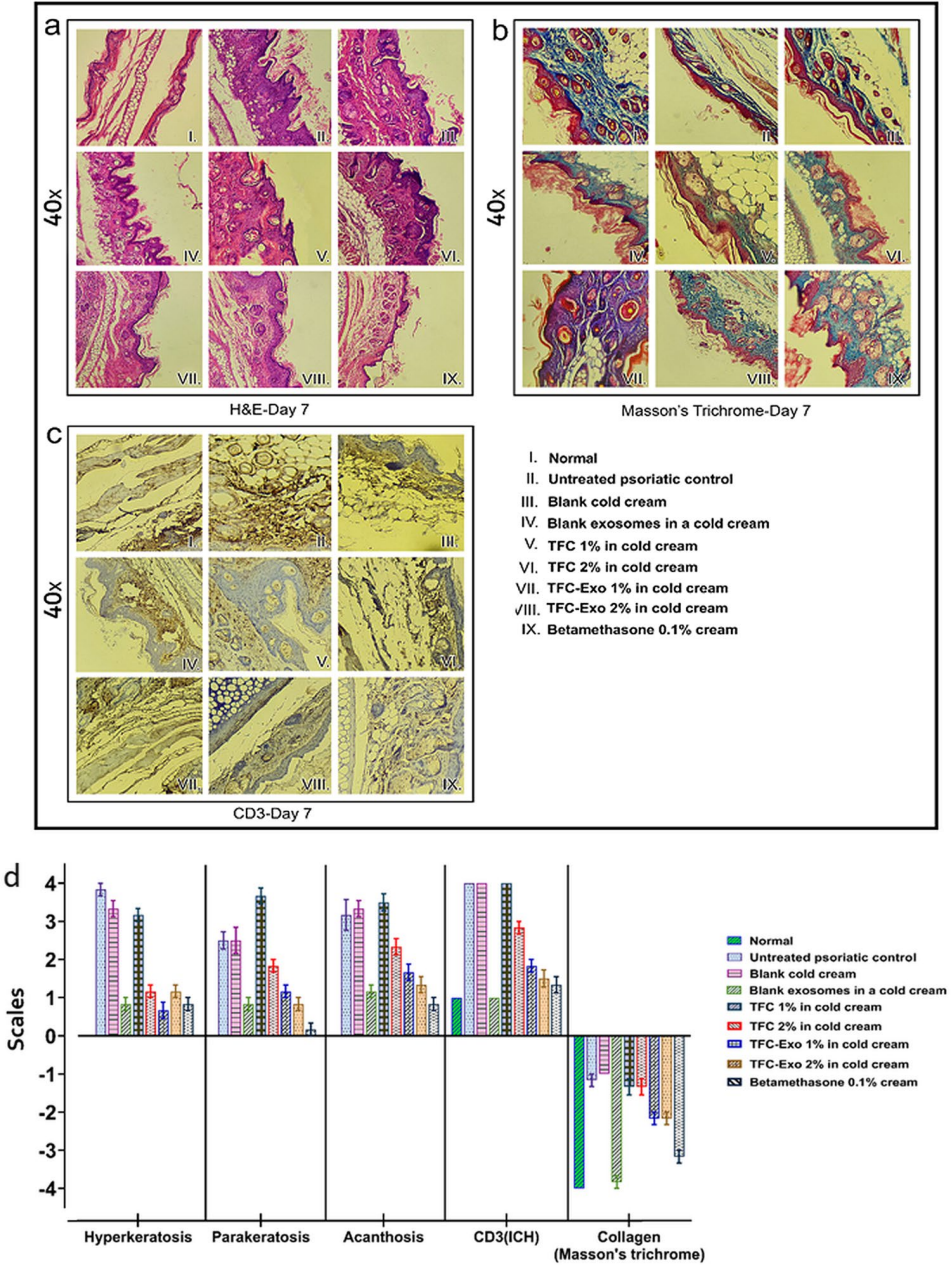
Microscopic images of animal ear tissues **a)** stained with H&E, **b)** Masson's trichrome, **c)** ICH (CD3) under a light microscope at 40× magnification and **d)** semi-quantitative assessments of histopathological results (mean±SD, n=6).

On the other hand, to investigate the histopathological changes caused by IMQ in the different groups with H&E staining, the three parameters of hyperkeratosis, parakeratosis, and acanthosis were studied (Figure 8a). For Mason's trichrome staining, the amount of collagen in the tissues was observed (Figure 8b) and for immunostaining tests the amount of CD3 was studied (Figure 8c). Semi-quantitative evaluation of all these parameters was done by Image J Software from photos and the results were shown in Figure 8d. While the levels of hyperkeratosis, parakeratosis, acanthosis and CD3 are elevated by IMQ and their increment indicates the severity of psoriasis-like symptoms, tissue collagen reduction occurs by IMQ. For this reason, the results of collagen changes were reported in reverse direction (Figure 8d).

**Fig. 9:**
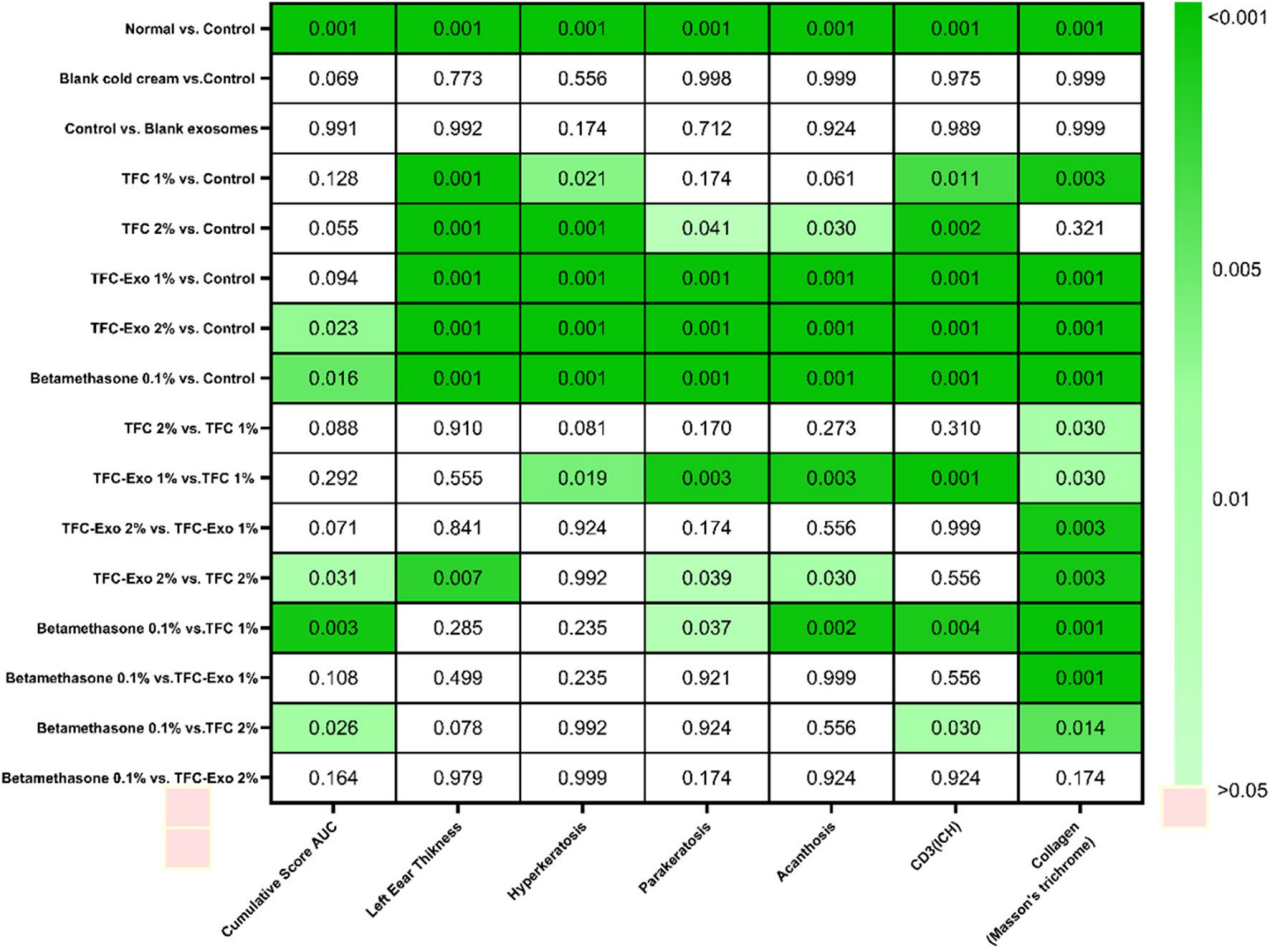
The *p* values of a pairwise comparison of studied groups.

Two macroscopic parameters of the left ear thickness changes and PASI and also the five histological parameters (hyperkeratosis, parakeratosis, acanthosis, CD3, collagen) were analysed using one-way ANOVA followed by Tukey's post-hoc test (Figure 9). Based on the results, the difference between the normal group and untreated psoriatic control group was statistically significant for all parameters (p <0.001). When untreated psoriatic control group was compared with blank cold cream group and blank exosomes in cold cream group, there were no significant changes in any of the parameters.

The *p* value of the examined parameters decreased when the untreated psoriatic control group was compared to the TFC 1%, TFC 2%, TFC-Exo 1%, TFC-Exo 2%, and Betamethasone 0.1% groups. Clearly, as the *p* value decreased, the investigated parameters returned to normality (similar to the conditions of the normal group).

In a large number of the examined parameters, there were no significant differences between free TFC 1% and 2% groups, or between the TFC-Exo 1% and 2% groups. This demonstrates the poor impact of increasing the therapeutic dosage on improving the inflammation induced by IMQ. However, when the free TFC 1% and TFC-Exo 1% groups, as well as the free TFC 2% and TFC-Exo 2% groups, were compared, significant changes were observed in five of the seven examined parameters (hyperkeratosis, parakeratosis, acanthosis, CD3 and collagen) for free TFC 1% *vs* TFC-Exo 1% and (left ear thickness AUC, cumulative score AUC, parakeratosis, acanthosis and collagen) for free TFC 2% *vs* TFC-Exo 2%.

As a result, in both *in vivo* and *in vitro* experiments, we found that TFC loaded in exosomes improved the pathogenesis and symptoms of psoriasis more than free TFC. However, the effect of blank exosomes on the increase of psoriasis symptoms relative to the control group was not observed significantly to the same extent as in the Real-time PCR study (Figure 5).

## Discussion

Many research works have been conducted on the influence of exosomes on cell communication, and hypotheses continue to emphasize the relevance of these communications in the development of psoriasis symptoms and pathogenesis. These interactions between keratinocytes and specialized immune cells are complicated in the etiology of psoriasis [10].

Several exosome isolation techniques are employed depending on the type of aims and applications needed. Ultracentrifuge, size-exclusion chromatography, polymer precipitation, and immuno-affinity techniques are the most often utilized [21]. All these techniques, however, have inherent limits. As the utilization of exosomes increased, new commercial kits were developed. These kits do not need specific equipment, so non-industrial labs with limited facilities and no requirements for large-scale production may be utilized. Isolation of exosomes from A-431 cells was the first step in the present research, followed by determining the best method for loading TFC into exosomes, identifying significant factors, and selecting the optimal formulation based on statistical analysis of the results.

TFC-Exo mean particle size, polydispersity index (PDI), and zeta potential were determined by DLS and play critical roles in determining the potential of the nano carriers for controlled drug release, *in vivo* biodistribution, and their stability. All methods except the freeze-thaw cycle produced TFC-Exo with a narrow PDI (Table II) and monodispersity, which is important for keeping them stable in dispersion. In the case of lipophilic nanoparticles, particle size is critical; a colloidal solution with smaller exosomes not only makes colloidal systems more stable but also has a significant impact on the ability of TFC-Exo to penetrate the skin and mucous membranes. Exosomes diameter is dependent on some parameters including; the method of exosome isolation and the cell-line source of exosomes [22]. The polydispersity index (PDI) is a measure of particle size distribution. As this index tends to zero, it suggests that the particle size is more homogeneous; as it approaches one, the particle size becomes heterogeneous. Among the drug loading methods, the most significant increase in particle size and PDI were seen in the freeze-thaw cycles method (Table II). The aggregation of exosomes during the freeze-thaw cycles probably contributed to the increase in this index. It also appears that more intensive methods increase these parameters. Studies show by increasing the number of lipophilic substances in nanoparticles the PDI is increased too [23].

While the zeta potential was significantly higher in the TFC-Exo produced by probe sonication method compared to the other drug loading methods (Table II), there was no significant difference between the zeta potential of the blank exosomes and TFC-Exo. Some papers and guidelines mention that the absolute value of zeta potential larger than 15 mV denotes a stable colloidal system, and this is particularly noticeable for developing drug delivery systems [24]. The stability of a colloidal solution is determined by forces of electrostatic repulsion and van der Waals attraction. If the van der Waals forces are weak, a lower zeta potential than usual may also be present in very stable colloidal systems [25]. Exosomes PEGylation has been used for their isolation by making them heavier for sedimentation after centrifugation. Previous researches have shown that PEGylation of nanoparticles, improves their colloidal stability despite decreasing the zeta potential [26].

Loading efficiency (LE%) and release efficiency (RE%) are two additional critical factors for choosing the optimal formulation. Although DLS studies and stability in colloidal dispersions are undeniably important, the percentage of loading efficiency (LE%) is the most important parameter among the examined factors for the selection method of loading TFC in exosomes due to economic concerns and saving in material and drug consumption, as well as giving researchers the choice to determine the appropriate therapeutic dosage.

In light of the studies done on release efficiency, it should be mentioned that when free TFC and TFC-Exo were added to the cold cream formulation, their release profiles changed fundamentally different from the TFC-Exo (Figure 3b). The drug release from exosomes was best fitted with a first order kinetic, while in creams it showed a Higuchi release kinetic model (Figure 3b). However, as the limitation of this study it may be considered just using the correlation coefficients of curve fitting to different kinetics models, while it was better to use Akaike Information Criterion (AIC) also for determining the release mechanism statistically by DDSolver Software. This criterion was not considered due to lack of access to the software.

Based on the results of zeta potential, loading efficiency, and release efficiency analysis, probe sonication was chosen as the best method for preparing TFC-Exo (Table II, Figure 3). These exosomes were then incorporated into a cold cream formulation for *in vivo* animal studies.

The MTT assay revealed that blank exosomes, were not cytotoxic to cells. The 8-hour sustained release of the drug from the exosomes in the 24-hour MTT test indicated a significant decrease in the cytotoxicity of TFC-Exo compared to free drug (Figure 4a) probably due to slow release of TFC from the exosomes (Figure 3a). This difference in cytotoxicity was most noticeable in the 24-hour MTT assay and IC_50_ results (Figure 4). However, there was no difference between the cytotoxicity of free TFC and TFC-Exo at 48 hours (Figure 4b) probably due to the release of more than 80% of the loaded drug from exosomes during 48 hours, which causes practically no difference with the free drug. Meanwhile, at the same 24-hour RT-PCR results on the same cell line, the anti-inflammatory activity of TFC-Exo was significantly greater than that of free TFC in all samples (Figure 5). RT-PCR examinations were done about the concerning the effects of blank exosomes and TFC-Exo on epidermoid carcinoma A-431 cells to gain a better perspective of the relevance of their interactions and the effect of exosomes on them. The selection of this cell line was motivated by several research on the existence and efficacy the cytokines involved in the JAK/STAT signaling pathway in A-431 cells, which is the primary mechanism of the TFC action on psoriasis [27].

In previous research, lesions on the skin of IMQ-induced psoriasis-like mice have been compared to human psoriasis lesions. The common symptoms were classified into macroscopic and histopathological categories. IMQ-induced psoriasis-like mice studies in recent years have followed relatively consistent recipes. However, depending on the type of study, there may be some changes. IMQ is often applied simultaneously to the shaved back and ears of the animal. In the first macroscopic study to simulate the psoriasis area and severity index (PASI) in the mouse model, three lesions of desquamation or scaling, induration or thickness, and erythema of the skin on the back of the mouse were measured every day as part of an objective rating system. In the second macroscopic study, the increase in the thickness of the treated left ear in each group of animals was measured compared to the first day. This increase in thickness indicates keratinocyte proliferation, the main symptom of psoriasis skin lesions [28]. Of course, in this study, due to the large number of studied groups, we measured the area under the curve (AUC) of each group after drawing the graphs of the macroscopic studies. In addition to these macroscopic parameters, five immunohistopathological parameters were also investigated. In the H&E stained samples, we evaluated the parameters of hyperkeratosis, which is evidenced by the increase in the thickness of the stratum corneum layer, parakeratosis, which is characterized by the observation of nucleated cells in the stratum corneum layer and is a sign of incomplete and prematuration of keratinocyte cells, and acanthosis, which is associated with excessive protrusion of Rete Ridge, infiltration of neutrophils inside the epidermis, and dilation of blood vessels. In the samples stained with masson's trichrome, the amount of tissue collagen can be seen as a blue color, which increases with the increase in healing. The last parameter is related to CD3 immunohistochemical evaluation, which shows the level of CD4+ and CD8+ infiltration. In total, these seven parameters can be used to compare animal groups with a reasonable degree of accuracy in terms of the efficacy of the administered treatments [29, 30].

In spite of our expectation that the results of RT-PCR on A-431 cells may be considerably different from the animal studies due to the suppression of inflammatory cascade pathway outside the keratinocytes and perhaps in immune cells, but they even showed clearer and more reliable results (Figure 5). This may demonstrate that most of the inflammatory interactions will happen in the keratinocytes themselves, even though they have an effect on other cells, especially immune cells [31, 32].

Another hypothesis is that because the studied exosomes were isolated from human keratinocytes, their surface biomarkers act more specifically on the human cell surface ligands. This is because the effect of exosomes on the same type of cells from which they were extracted was stronger in the RT-PCR results than in the animal studies, in which the effect of the human keratinocyte-derived exosomes was studied on the mouse model of disease.

Psoriasis is characterized by abnormal activation of the immune system, and hyperproliferation and aberrant differentiation of keratinocytes. Psoriatic keratinocytes death is also recently recognized as a major amplifier to the initiation of inflammatory cascade. Given that both keratinocytes and immune cells express high PD-1 in psoriasis, which imply PD-1 as a potential therapeutic target for psoriasis. Jia *et al*. [33] reported that HaCaT keratinocytes, the tumor-derived PD-L1 + exosomes, have a natural inflammatory tropism and excellent anti-inflammatory effect, and able to act as a bio-inspired nanocarrier for various therapeutic agents to optimized inflammatory disease therapy. Therefore, a strength of our study is the use of exosomes shedded from A-431 cells, which are human epidermoid carcinoma cell line and can target the drug to the inflamed areas involved with psoriasis. However, a drawback of using these cells as the source of exosomes may be the potency of these tumor derived exosomes which may play an important role in cancer metastasis by altering tumor microenvironment through modulating cell-cell communication between tumor derived exosomes and distant host cells [34]. Although we used exosomes of keratinocytes with a source of cancer cells, but because of controversial reports, still exosomes of different cell lines like macrophages, mesenchymal stem/stromal cell-derived exosomes, umbilical cord-derived mesenchymal stem cell-derived exosomes and other sources should be tested to obtain the most efficient exosomal formulation, considering the different results that may be obtained for the shedding of exosomes, drug loading efficiency, the release efficiency of cargo, and therapeutic anti-psoriatic effects.

## Conclusions

We showed in this study, the use of exosomes as novel carriers in targeted drug delivery of TFC for treatment of psoriasis, because of the specific biomarkers on their surface and their role in communication between particular cells. It can help us in diseases where communication between cells is the determining factor in the disease. In addition to the role of communication between keratinocyte cells and other nearby cells such as blood cells, we found out the very wide and undeniable role of keratinocyte cells themselves in increasing the production of related cytokines in the flare-up of psoriasis. It is suggested to design a comprehensive study to compare the effects of communication of different cell lines involved in psoriasis. The role of drug encapsulation in exosomes was shown *in vivo*. This method of drug delivery for TFC may be effective on enhancing its therapeutic effects and reduction its side effects favorably in chronic administration.

## Data Availability

Data are available on request.

## Declarations

### Research involving Animals

*All animal experiments complied with the ARRIVE guidelines and were carried out in accordance with the National Institutes of Health guide for the care and use of Laboratory animals (NIH Publications No. 8023, revised 1978).* The guidelines for animal research established by the Isfahan University of Medical Sciences ethical committee were followed (ethical code: IR.MUI.RESEARCH.REC.1399.589), which were according to the NIH Guide for the Care and Use of Laboratory Animals.
